# 837 Impact of Autologous, Non-Autologous, Synthetic Tissue Substitutes in Burns 2016-2021: Analysis of National Burn Repository

**DOI:** 10.1093/jbcr/iraf019.368

**Published:** 2025-04-01

**Authors:** Roselle Crombie, Claire Witherel

**Affiliations:** Connecticut Burn Center, Bridgeport Hospital / Yale-New Haven Health; Integra LifeSciences

## Abstract

**Introduction:**

Over 75 commercially available technologies, skin substitutes (aka cellular and acellular matrix-based products, CAMPs), have been used to manage thermal injuries over the last 20 years. Despite demonstrated long term safety and efficacy, the use of CAMPs remains controversial, in terms of clinical benefit and economics. Until recently, very few clinical studies have investigated skin substitute use product-agnostically. The goals of this study are to investigate groups of CAMPs use for burns, specifically, autologous, non-autologous tissue substitutes, and synthetic tissue substitutes and the impact of their use in the burn care from 2016-2021.

**Methods:**

A subset of the National Burn Repository data from 2016-2021 were analyzed (n =118,928 patients). Patients treated with an autologous only (n=22,074), non-autologous only (n=21,058), synthetic only (n=2701), non-autologous and autologous (n=11,996), synthetic and autologous (1,296) tissue substitutes, or none (n=56,644) of those during their care were identified via ICD-10 codes.

**Results:**

Preliminary analysis of majority third-degree burn patients that were managed with non-autologous or synthetic tissue substitutes alone or in combination with an autologous tissue substitute showed that there were no significant differences between non-autologous and synthetic tissue substitutes with respect to LOS/TBSA, days on a ventilator, days in the ICU, complications, number of resources, or number of overall procedures regardless of TBSA bucketing (10-19%, 20-39%, 40-59, & >60%). There were also observed inconsistencies between patients treated with specific ‘resources’ and the procedure codes.

**Conclusions:**

This study illustrates the first analysis of the NBR to investigate specific care algorithms based on procedures and resources utilized in burns. In addition to CAMP use being controversial in practice, there is disagreement on which type of CAMP to use when, where, and how they are coded. This study begins to retrospectively elucidate outcomes associated with one care pathway over the other. Future analysis will propensity match patient cohorts to understand the clinical and health economic implications of CAMP substitute use in burns.

**Applicability of Research to Practice:**

Given austere resource utilization, supply chain issues and ongoing challenges long after COVID, its important to start to elucidate the role and impact of CAMPs in burn care.

**Funding for the Study:**

Acquisition of a short subscription of Bdata was obtained by Integra Life Sciences. Analysis and research was not funded.

